# Tonic GABA_A_ Receptors as Potential Target for the Treatment of Temporal Lobe Epilepsy

**DOI:** 10.1007/s12035-015-9423-8

**Published:** 2015-09-26

**Authors:** S. Schipper, M. W. Aalbers, K. Rijkers, A. Swijsen, J. M. Rigo, G. Hoogland, J. S. H. Vles

**Affiliations:** 1 Department of Neurology, Maastricht University Medical Center, Maastricht, The Netherlands; 2 Faculty of Health Medicine and Life Sciences, School of Mental Health and Neuroscience, Maastricht University Medical Center, Maastricht, The Netherlands; 3 Department of Neurosurgery and Orthopedic Surgery, Atrium Hospital Heerlen, Heerlen, The Netherlands; 4 BIOMED Research Institute, Hasselt University/Transnational University Limburg, Martelarenlaan 42, 3500 Hasselt, Belgium; 5 Department of Neurosurgery, Maastricht University Medical Center, Maastricht, The Netherlands

**Keywords:** Extrasynaptic GABA_A_ receptor, Epilepsy, Seizures, Tonic, Antiepileptic drugs

## Abstract

Tonic GABA_A_ receptors are a subpopulation of receptors that generate long-lasting inhibition and thereby control network excitability. In recent years, these receptors have been implicated in various neurological and psychiatric disorders, including Parkinson’s disease, schizophrenia, and epilepsy. Their distinct subunit composition and function, compared to phasic GABA_A_ receptors, opens the possibility to specifically modulate network properties. In this review, the role of tonic GABA_A_ receptors in epilepsy and as potential antiepileptic target will be discussed.

## Introduction

Worldwide, more than 50 million people are suffering from epilepsy [1]. Temporal lobe epilepsy (TLE) is the most common type of partial onset epilepsy [2]. Epilepsy has a severe impact on patients’ quality of life, first, because patients experience unpredictable seizures that restrict them in activities of daily living and second, because patients can suffer from several neuropsychiatric comorbidities such as depression or cognitive decline. Current treatment options for epilepsy are insufficient. Approximately 30 % of patients are drug resistant [3], which is defined as failure to achieve seizure freedom despite two tolerated, adequately applied antiepileptic drug (AED) schedules [4]. Furthermore, AEDs can have side effects, including somnolence, behavioral changes, dizziness, and weight gain. Therefore, there is an urgent need for more efficacious AEDs with fewer side effects.

The γ-aminobutyric acid (GABA) type A receptor (GABA_A_R) is an important target for AEDs, as it is the most important inhibitory receptor in the central nervous system and therefore plays an important role in the development and maintenance of epilepsy. Upon binding of the neurotransmitter GABA to the ionotropic GABA_A_R, the receptor opens allowing chloride and bicarbonate ions to diffuse into the cell. This results in hyperpolarization and a higher excitation threshold, i.e., an inhibitory postsynaptic potential (IPSP) [5, 6]. GABA_A_Rs mediate two different types of inhibition: phasic inhibition characterized by a short-lasting IPSP and tonic inhibition characterized by persistent, long-lasting IPSP. GABA_A_Rs mediating tonic inhibition are different from those mediating phasic inhibition. They are located outside the synapse and hence are referred to as perisynaptic or extrasynaptic receptors. Moreover, the subunit composition of tonic GABA_A_Rs differs from that of phasic GABA_A_Rs.

Due to its long-lasting hyperpolarization, tonic inhibition can be considered as a constant “brake on the system” counterbalancing excitation [7]. Tonic currents are therefore involved in a broad array of vital physiological functions, such as regulating neuronal excitability, network oscillations, synaptic plasticity, neurogenesis, neuronal development, information processing, and cognition [5, 8–14]. The importance of this type of inhibition is further emphasized when realizing that under physiological conditions, the charge carried by tonic GABA currents is bigger than that of synaptic currents. For instance, tonic inhibition is responsible for generating 75 % of the total inhibitory charge received by hippocampal neurons [15].

Considering the important role of tonic GABA signaling in regulating network excitability, alterations in tonic signaling must play a role in epilepsy. The current review discusses physiological tonic GABA signaling, its role in TLE, and describes existing and future strategies using tonic currents as novel targets for antiepileptic treatment.

## Physiology of Tonic GABA Signaling

In order to discuss the pathophysiological changes taking place in epilepsy, it is first necessary to understand the physiological principals governing tonic signaling in the central nervous system. The following paragraphs will discuss the molecular composition of GABA_A_Rs, determinants influencing tonic signaling including the role of GABA_B_ receptors (GABA_B_Rs), the specific role of tonic inhibition in interneurons, and clustering of GABA_A_Rs.

### Subunit Composition of Tonic GABA_A_Rs

GABA_A_Rs are heteropentamers assembled from α1-6, β1-3, γ1-3, δ, ε, π, and θ subunits [16, 17]. In theory, these different subunits can combine into thousands of different GABA_A_Rs. In reality however, GABA_A_Rs have preferential configurations, which means that the number of different GABA_A_Rs expressed in vivo is limited. Mostly, two α subunits combine with two β subunits and either a γ or a δ subunit [18]. Some subunits are inhomogeneously distributed over the brain [19, 20]. For instance, α5 is predominantly found in the hippocampal dentate gyrus (DG) and cornu ammonis (CA) 1 and 3 region. In contrast, δ and γ subunits are found in the cortex, hippocampus, thalamus, striatum, and cerebellum. At the subcellular level, some subunits and subunit combinations are preferentially expressed at extrasynaptic sites (Table 1). For instance, α5 subunits and receptors consisting of an α4, α6, and δ subunit are predominantly expressed at extrasynaptic sites [21, 22], whereas, γ2 is mostly expressed at the synaptic site [7, 9, 21, 23–27]. To date, knowledge of the processes guiding these preferential expression sites is incomplete. In general, it is assumed that guidance is driven by the interaction between subunits, anchoring proteins and the cytoskeleton [28].

**Table 1:** Subunit composition of extrasynaptic GABA_A_Rs depends on brain region and cell type

GABA_A_R subunits	Structure	Cell type
α5βγ	Subiculum	Pyramidal cells [16]
		Interneurons [17]
	Hippocampus	Granular cells [18]
	CA1	Pyramidal cells [7]
α6βδ	Cerebellum	Granular cells [19–21]
	Hippocampus	Pyramidal cell [7]
α4βδ	Thalamus	Relay neuron [22–26]
	Hippocampus	Granular cells [18, 20, 27, 28]
	Frontoparietal cortex	Pyramidal cells [29]
α1β2δ	Hippocampus (Molecular layer)	Interneurons [25]
α3βγ	Basolateral amygdala	Pyramidal cells [30]
α5β3γ2	Striatum	Juvenile D1+/D2+ cells [31, 32]

In addition to a specific distribution, the subunit composition of GABA_A_Rs also influences the affinity for GABA. For instance, receptors containing an α3 subunit have a higher affinity for GABA than α5 containing GABA_A_Rs [29–32]. Similarly, δ subunit containing GABA_A_Rs have a higher affinity than those expressing a γ subunit. This implicates that extrasynaptic GABA_A_Rs have a higher affinity for GABA than synaptic ones [5, 33]. Finally, the subunit composition can also affect the kinetic properties of GABA_A_Rs, e.g., δ subunit containing GABA_A_Rs desensitizes slower [5].

### Activation of Tonic GABA_A_Rs

**Fig. 1:**
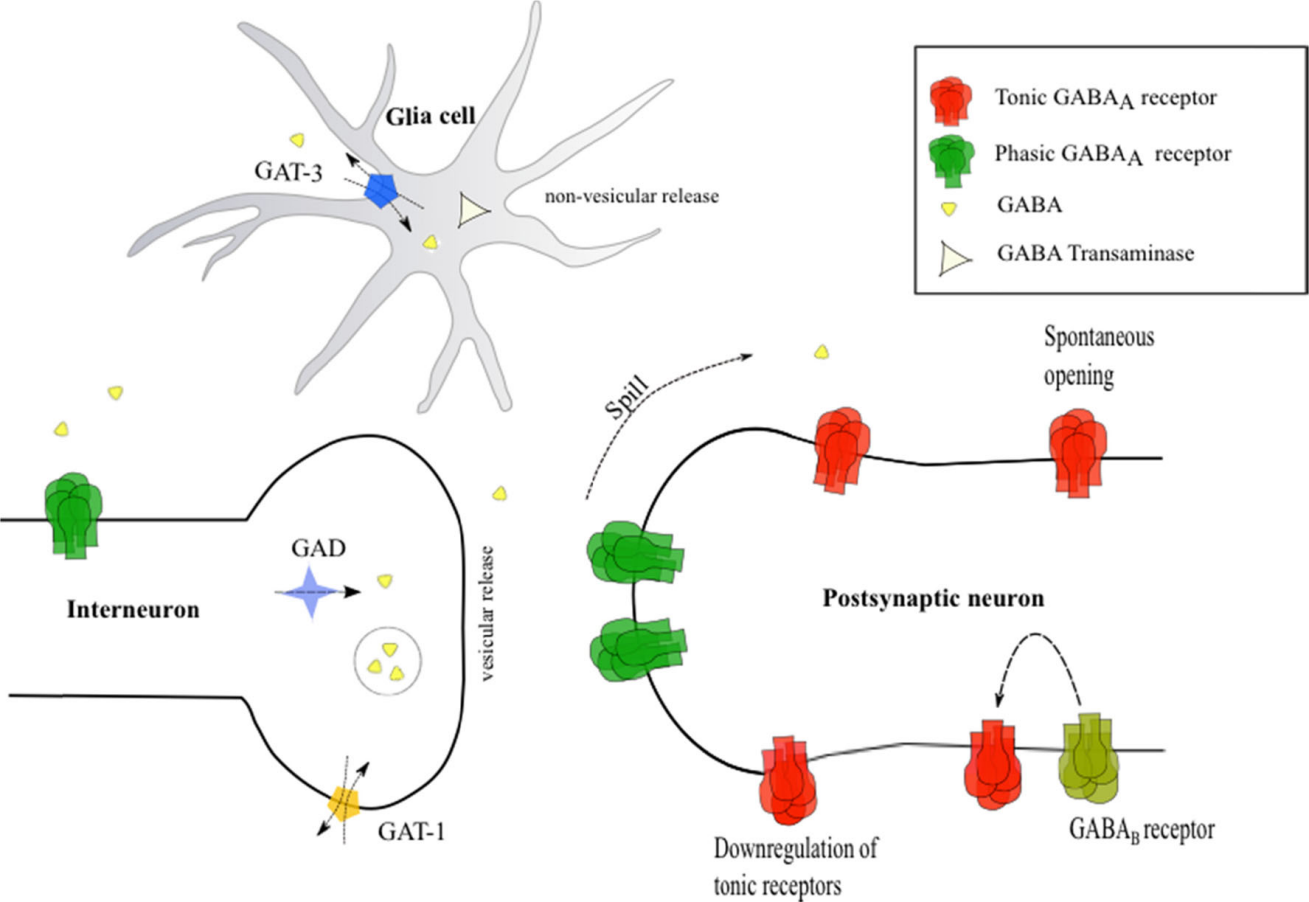
Mechanisms modulating the activity of tonic GABA_A_Rs. Upon release into the synaptic cleft, GABA is taken up in glia cells by GABA transporter (GAT) type 3 and metabolized by GABA transaminase or taken up presynaptically by GAT-1. Tonic GABA_A_Rs can be activated by four mechanisms: (1) spill of synaptically released GABA into the extrasynaptic space due to insufficient clearance by GATs, (2) non-vesicular GABA release by GAT reversal, (3) spontaneous opening in the absence of extracellular GABA, and (4) GABA_B_ receptor activation increases tonic GABA_A_R signaling via an intracellular mechanism

Tonic GABA_A_Rs can be activated in several ways (Fig. 1). First of all, repetitive activation of the presynapse results in increased levels of GABA in the synaptic cleft. This is due to insufficient time for clearance by GABA transporters (GATs). As a consequence, GABA can accumulate in the synaptic cleft. Consequently, GABA diffuses to the extracellular space where it activates extrasynaptic receptors [21, 34, 35]. The degree and speed of diffusion are influenced by parameters such as the distance between the synaptic cleft and receptor, diffusional barriers, and geometry of the synapse [36].

Second, tonic GABA_A_Rs may be activated by GABA that is released from non-vesicular sources [37, 38]. Extracellular GABA may come from glial cells or dendrites (for a review, see Yoon and Lee [39]) [40–44], for instance by GAT reversal [45–51].

Third, opening of tonic GABA_A_Rs can occur spontaneously in the absence of synaptic activation and even in the absence of extracellular GABA [52, 53]. This has been shown in vitro for β1 subunit containing GABA_A_Rs in the rat [54], the human α1β3ε receptor [55], human α1β1 and α1β1γ2 receptors [56], and in rat hippocampal slices [57].

The three proposed mechanisms of tonic GABA_A_R activation might be complementary: In case of baseline network activity, tonic activation may be caused by non-vesicular sources of extracellular GABA or by spontaneous opening, while in case of excessive network activity, GABA may spill into the extracellular space.

The magnitude of tonic signaling is influenced by several factors, such as the amount of ambient GABA and the availability of receptors. Moreover, neuromodulators can alter tonic GABA signaling. An example of neuromodulators are neurosteroids, a group of neuromodulators that influences δ subunit containing tonic GABA_A_Rs. Binding of these neurosteroids increases tonic inhibition. Next to this acute effect, neurosteroids also cause downregulation of certain subunits such as the γ2 and δ subunits in the DG [58]. Consequently, tonic and phasic currents decreased [59].

The mechanisms governing tonic inhibition are summarized and illustrated in Fig. 1.

### The Influence of GABA_B_ Receptors on Tonic GABA Signaling

Metabotropic GABA_B_Rs are expressed both, presynaptically and postsynaptically, and are known to modulate seizure activity. At the presynapse, they act as autoreceptors, i.e., activation of this receptor causes a reduced Ca^2+^ entry, resulting in a decreased GABA release. At the postsynaptic site, GABA_B_Rs can modulate tonic signaling. For instance, activation of GABA_B_Rs by baclofen increases the tonic current of δ containing GABA_A_Rs in thalamocortical cells, DG, and cerebellar granule cells [60]. The mechanisms by which GABA_B_ signaling controls tonic GABA_A_R currents are not fully understood. It is hypothesized that activation of postsynaptic GABA_B_Rs initiates a G-protein coupled signal transduction to δ containing GABA_A_Rs (for review, see [61]). The contribution of this pathway versus that of autoreceptors in the proconvulsive or anticonvulsive activity of GABA_B_R-modulating drugs remains to be elucidated.

The spatial expression of GABA_B_Rs at the postsynaptic membrane shares striking resemblance with that of extrasynaptic or perisynaptic GABA_A_Rs. Most likely GABA_A_Rs and GABA_B_Rs are activated simultaneously due to spillover of GABA [34, 62, 63]. Furthermore, GABA_B_Rs are found on astrocytes. As these cells have an essential buffering capacity, glial GABA_B_Rs may be important in regulating the concentration of extracellular GABA [64, 65].

### Clustering and Trafficking of GABA_A_R

**Fig. 2:**
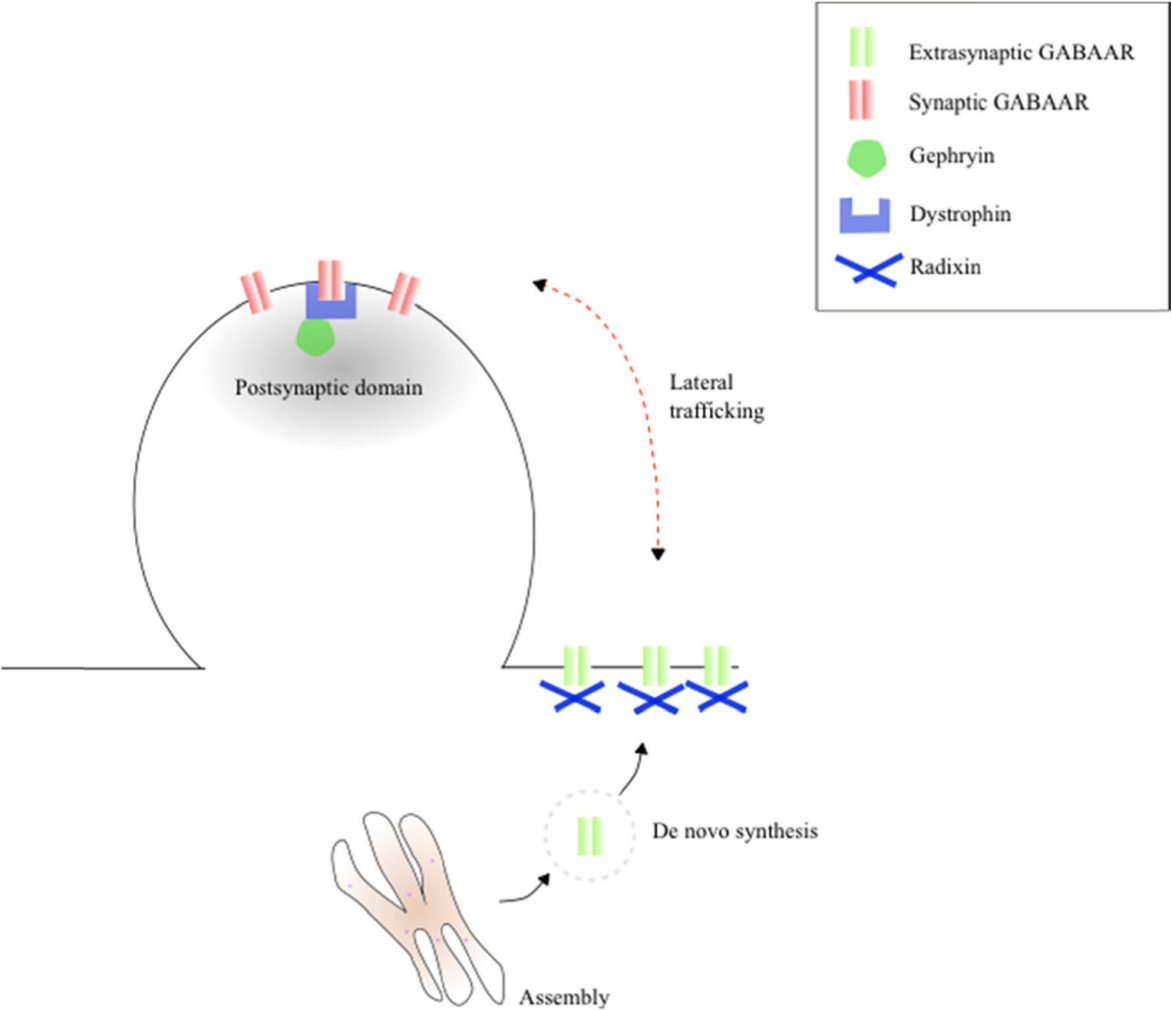
Receptor trafficking to the membrane and between the extrasynaptic and synaptic site. Additionally, examples of extrasynaptic and synaptic anchoring proteins are shown

The expression of GABA_A_Rs is tightly controlled and depends on assembly, maturation, and recycling of different subunits. These processes are regulated by a complex interaction of various proteins such as GABA_A_R-associated protein (GABARAP) and N-ethylmaleimide-sensitive factor (for a review, see Lorena Arancibia-Cárcamo 2009). Normally, GABA_A_Rs are inserted into the cell membrane at the extrasynaptic location and diffuse via lateral trafficking into the postsynaptic density (PSD; Fig. 2). Here, they are clustered by different adhesion and scaffolding proteins, i.e., gephryin, dystrophin, and neurexin [66]. GABA_A_Rs can also be clustered at the extrasynaptic site by molecules such as radixin [67, 68]. Receptors are able to migrate back and forth between the synaptic and extrasynaptic site [69, 70] and can rapidly cycle back after endocytosis to the extrasynaptic membrane [28]. It is unclear which mechanisms cause receptors to diffuse away from the synaptic site or facilitate their entry into the PSD.

An increase in extrasynaptic clustering influences tonic signaling. Surprisingly, not only the amount of channels but also individual channel properties change with increased clustering [71, 72]. Clustered GABA_A_Rs have a higher EC_50_, deactivate faster, and desensitize slower compared to diffuse receptors [72]. The heterogeneity of receptors and preference of certain subunits to cluster extrasynaptically complicate the understanding of this mechanism [71].

### Tonic Inhibition in Interneurons

The activity of interneurons paces the hippocampal rhythm. This rhythmogenesis is therefore partly controlled by the tonic GABA conductance in interneurons. This is demonstrated by the finding that δ subunit knockout mice show higher frequency γ oscillations [11]. However, interneurons show a bivalent response to ambient GABA. At low levels of GABA, tonic inhibition is reduced, causing an enhanced excitability of interneurons [24, 73] and consequently an increased inhibition of pyramidal cells. Moreover, this weak tonic conductance imposes regular firing of interneurons that synchronizes the CA 3 network [74]. On the other hand, high concentrations of ambient GABA inhibit interneurons [75]. As a result, they become less excitable and thus release their brake on pyramidal cells. However, under these high extracellular GABA levels, the pyramidal cells themselves also receive more tonic inhibition. This inhibition can counterbalance the loss of interneuronal inhibition. Thus, control of the extracellular GABA concentration provides both a direct and indirect mechanism to regulate pyramidal cell activity [73].

## Tonic GABA Signaling in Temporal Lobe Epilepsy

Considering the important role of tonic GABA signaling in regulating network excitability, it is likely that tonic GABA signaling is altered in the occurrence of seizures or in the development of a seizure prone network (epileptogenesis) [76, 77]. Studies on tonic GABA signaling in epilepsy will be discussed in the following paragraph and are summarized in Table 2.

**Table 2:** Summary of the evidence concerning the role of subunit expression in temporal lobe epilepsy

Author	Year	Model	Species	Technique	Cell type	Decrease	Increase	Additional findings
Bouilleret	2000	SE KA i.c.	Mouse	IHC	DG (GC/ML) CA1	α5, γ2	γ2, α5, α1	Loss of GAT-1 in CA1 and DG, not in CA3
Brooks-kayal	1998	SE Pilocarpine	Rat	Whole cell patch clamp, Single-cell mRNA amplification	DG	α1 (E)	α4, δ	Altered sensitivity to zolpidem and zinc Increased GAD67 expression
Drexel	2013	SE KA	Rat	In situ hybridization	DG CA1 CA3	α5, δ γ2 (E), δ α5, γ2 (E)	α4, α1 α1	
Fritschy	1999	SE Pilocarpine	Rat	IHC	DG (GC/ML) CA3	α1 α5	α3, α5 γ2	
Goodkin	2008	SE Continuous hippocampal stimulation	Rat	Whole cell patch clamp (DG)				Maintenance of tonic GABA currents No reduction of δ subunit expression
Houser	2003	SE Pilocarpine	Rat	IHC In situ hybridization	CA1/2	α5		
Kamphuis	1995	Amygdala kindling	Rat	In situ hybridization	DG (GC)	γ2 (L)	α1/2/4 (E), γ2 (E)	
Lee	2013	SE Pilocarpine	Rat	Whole cell patch (DG) 2–3 weeks after status epilepticus				Increase tonic inhibition in GC in DG
Loup	2000	Human	Human, hippocampal sclerosis	IHC	DG GC ML		α1, γ2 α2	
Naylor	2005	SE Pilocarpine	Rat	Whole cell patch clamp (DG)				Increase in tonic GABA_A_R mediated currents one hour after SE
Nishimura	2005	SE Hippocampal kindling Self-sustained limbic status epilepticus	Rat	In situ hybridization	DG (GC) CA1 CA3	α5 (E/L), δ (E/L) α5 (E/L) α5 (E/L), γ2 (E)	γ2 (E)	
Peng	2004	SE Pilocarpine	Mouse	IHC	DG (ML) DG (IN)	δ	δ	
Rajasekeran	2010	SE Continuous hippocampal stimulation	Rat	Patch clamp Western blot	DG	δ	α4	Tonic currents are maintained in DG cells post-SE by α4γ2 receptors Reduced neurosteroid (allopregnanolone, L655708) sensitivity in epileptic DGC Retention of δ subunit in ER
Scimemi	2005	SE Pilocarpine/KA	Rat	Whole cell patch clamp CA1 IHC	CA1/3	α5		Maintenance of tonic GABA currents
Schwarzer	1997	SE KA	Rat	IHC	DG (ML)	α2, δ (E)	α1/2/4/5, δ, γ2 (L)	
Sun	2013	i.c. CTZ injection	Cell culture Rat	Whole cell patch clamp in cultured hippocampal neurons overexpressing α5β3γ2 and α6β3δ Field potentials in vivo				Overexpression α5β3γ2 and α6β3δ resulted in enhanced tonic inhibition and reduced epileptiform activity in vitro THIP (5 μM) suppressed epileptiform burst activity and behavioral seizures
Tsunashima	1997	SE KA	Rat	In situ hybridization	DG (GC) CA1 CA3	α5, γ2 (E) δ (L) α5, γ2 (L) α5, γ2		
Zhan	2009	SE Pilocarpine	Rat Mouse	Whole cell patch clamp (DG)				Increase in tonic signaling Tonic currents are maintained by α4βxδ and α5βxγ
Zhang	2007	SE Pilocarpine	Mouse	Immunogold-electronmicroscopy Whole cell patch clamp	DG (GC)	δ		Shift of γ2 toward perisynaptic location Magnitude of tonic GABA currents maintained

*DG* hippocampal dentate gyrus, *E* early, *GC* granular cell, *i.c.* intracerebral, *IHC* immunohistochemistry, *IN* interneuron, *KA* kainic acid, *ML* molecular layer

Single nucleotide polymorphisms and mutations in genes coding for tonic subunits are associated with several types of epilepsy [78–80]. For instance, Dibbens et al. [78] and Feng et al. [81] showed that genetic alterations in the GABRD gene, that codes for the tonic δ subunit, cause a decrease in tonic inhibition in complex idiopathic generalized epilepsies. Eugene et al. [80] found that in human epileptic syndromes with febrile seizures, mutations in the γ subunit cause a decrease in tonic currents by reducing the surface expression of α5 containing GABA_A_Rs.

Results on the expression levels of tonic subunits in experimental epilepsy are contradictory (for a full overview, see Table 2). Whereas several studies showed an increase of the α5 subunit [82–84] particularly in the DG in the kainate and pilocarpine model, others showed a decrease in CA1 in the pilocarpine model [84], in the DG in the kainate model [85] in CA1, CA2, and CA3 in the pilocarpine model [83], and in the DG, CA1, and CA3 in the hippocampal kindling model and the pilocarpine model [86–88]. The total and surface expression of the δ subunit in the DG of the hippocampus decreases after status epilepticus, both during the latent phase shortly after the induction of a status epilepticus and during the chronic phase, when animals experience spontaneous recurrent seizures [89–91]. As the expression of the δ subunit is concomitantly increased in the microsomal fraction, these results suggest that these subunits do not reach the cell membrane and are retained in the endoplasmatic reticiulum. All together, is seems that there is a quantitative decrease in the amount of tonic subunit expression at the messenger RNA (mRNA) and protein level in different hippocampal regions acutely after an epileptogenic insult but also during the chronic phase.

Nonetheless, electrophysiological studies have shown that alteration in the expression of tonic subunits is not accompanied by a functional loss of tonic inhibition [89, 91–93]. Some studies even report an increase of tonic signaling in experimental epilepsy shortly after status epilepticus and during the epileptogenic phase in the pilocarpine model [88, 94]. If, as animal studies suggest, subunits providing tonic currents are downregulated and tonic inhibition is unchanged or increased, then tonic GABA currents must be maintained by other means. What factors are possibly involved in maintaining tonic currents?

As the amplitude of tonic GABA signaling is determined by the concentration of GABA in the synaptic cleft, an increased GABA concentration could compensate for a decrease in the quantity of tonic receptor subunits. As discussed before, increased extracellular GABA concentrations can result from a reduced activity or number of GATs. Indeed, GAT-1, which is expressed presynaptically, was shown to be upregulated in the molecular layer of the hippocampus acutely after experimental status epilepticus, in the chronic phase in the kainic acid model [95] and in hippocampal specimen from patients with temporal lobe epilepsy [96]. Andre et al. [97] have shown that GAT-1 alterations depend on the time point in the epileptogenic process and the region investigated. Whereas GAT-1 was upregulated in the inner molecular layer of the hippocampus, it was downregulated in CA1 in rats with spontaneous, recurrent seizures compared to controls. Another important regulator of extracellular GABA is GAT-3, which is located on glial processes. The expression of both GAT-1 and GAT-3 are altered in hippocampi from TLE patients. As these changes vary per hippocampal subregion and GATs can reverse, causing non-vesicular GABA release, it is difficult to predict the effect of these alterations to hippocampal physiology [63, 95].

Whether an actual functional reversal of transporters occurs in epilepsy is difficult to establish due to the technical restrictions of measuring intracellular GABA concentrations. GAT reversal is eventually favored by an increased rate of GABA synthesis by GAD [95] that in its turn can be caused by an increase in metabolic rate of neurons as seen in epilepsy. Another possibility of increasing extracellular GABA is by increased activity of glutamic acid decarboxylase (GAD), the enzyme responsible for GABA synthesis. Esclapez and Houser [98] have shown that GAD 65 and 67 are increased in animals with spontaneous, recurrent epileptic activity at the mRNA and protein level. Acutely after status epilepticus, GAD 65 and 67 expression decreased in the hippocampal hilus, whereas GAD 67 expression in the DG is increased [99]. In the chronic phase, GAD accumulated in interneurons of the DG [82, 100]. Interestingly, GAD 65 null mice show spontaneous seizures [101].

Additionally, different channels (e.g., two-pore domain potassium channels) and receptors or GABA_A_Rs with a different subunit composition could take over tonic function [74, 102]. Indeed, pharmacological experiments demonstrated that increased tonic GABA currents are not mediated by α5- but δ-containing GABA_A_Rs in epilepsy [88]. In several studies, it has been shown that the downregulation of δ subunits in the DG is accompanied with an increase in the expression of receptors containing the α4 and γ2 subunits [86, 89, 90, 103]. As mentioned before, these receptors have a lower affinity for GABA, which implies that tonic GABA currents can only be maintained if there is an increase in extracellular GABA. Possibly, receptors that are typically expressed synaptically under physiological conditions are increasing shifted toward the extrasynaptic site in epilepsy. Evidence for this process comes from the shift of the γ2 subunit that is increasingly expressed at the extrasynaptic site in a murine model of epilepsy [104]. Additional electrophysiological studies are necessary to fully understand the functional consequences of quantitative changes in GABA_A_Rs.

## Toward Antiepileptic Drugs that Target Tonic GABA Signaling

Considering that tonic currents are functionally preserved in epilepsy, they constitute a potential treatment target [5, 15]. In order to increase tonic currents, there are two strategies: increasing the extracellular concentration of GABA or agonizing GABA_A_Rs by ligands binding to tonic subunits. In Fig. 1, the mechanisms modulating tonic signaling are displayed. These mechanisms will be discussed in the light of increasing tonic inhibition in the next paragraph.

### Increasing the Extracellular GABA Concentration

Extracellular GABA concentrations are controlled by synthesis, breakdown, and clearance from the synaptic cleft. The synthesis of GABA in the presynaptic terminal is determined by the activity of GAD 65, and the activity of single GAD is influenced by processes such as phosphorylation and the presence of co-factors such as pyridoxal 5′-phosphate. GAD concentrations can be increased by enhancing its transcription by stimulating promoter activity or by influencing epigenetic mechanisms that lead to a higher transcription rate [89, 105]. Alternatively, GAD can be potentiated by viral vector-mediated targeted delivery of therapeutic GAD. This strategy has already proven to be successful in experimental and clinical studies for Parkinson’s disease [106–108]. An advantage of viral vector-mediated targeted delivery therapy is its spatial specificity that potentially eliminates site effects caused by unspecific targeting.

Alternatively, a higher release of GABA could be achieved by increasing the concentration of GABA in individual presynaptic vesicles. GABA is packed into vesicles by vesicular neurotransmitter transporters (VGAT), and their activity depends on electrochemical components. Boosting vesicular GABA transport might be achieved by pharmacologically increasing the activity of VGAT or by increasing its gene expression.

A second mechanism to increase extracellular GABA concentrations is inhibiting breakdown of GABA that is mediated by GABA transaminase (GABA-T). The second-line AED vigabatrin works via irreversibly inhibiting GABA-T [109, 110], increasing the concentration GABA in the synaptic cleft [111]. Furthermore, there is evidence that vigabatrin increases GABA release and constrains glial GABA uptake [112, 113]. Other more potent and novel inhibitors of GABA-T are ethanolamine-O-sulfate (EOS), L-cycloserine, and phenylethylidenehydrazine. Interestingly, the latter is used in various psychiatric disorders and increase extracellular GABA concentrations in vivo and decrease epileptiform activity in the rat ex vivo [114]. However, its anticonvulsant properties in vivo varied depending on the seizure model used. Several other GABA-T inhibitors such as gabaculine, gamma-acetylenic GABA, and gamma-vinyl GABA do not appear suitable for treatment of convulsive disorders in humans due to their severe and sometimes lethal side effects [115].

The last mechanism to increase extracellular GABA concentrations is by decreasing GABA clearance from the synaptic cleft by influencing GAT. Two drugs acting at GATs are SNAP-5114 and tiagabine that inhibit GAT-2 and -3, and GAT-1, respectively [116]. Indeed, they increase the concentration of extracellular GABA in vivo [117]. Tiagabine seems to be efficient as an add-on treatment in partial and secondarily generalized seizures, reducing seizure frequency [118]. However, clinical data show disappointing results with patients suffering from paradoxical proconvulsive effects [119]. So far, no pharmaceutical interventions exits that acts by reversing GATs.

### Extrasynaptic GABA_A_R Agonists

In addition to enhancing extracellular GABA, enhancement of tonic GABA_A_Rs can also be achieved by using specific agonists. Ganaxolone is a neurosteroid and a tonic GABA_A_R agonist, binding to and influencing the δ subunit. At low concentrations, it potentiates GABA_A_Rs (positive allosteric modulator), while at higher doses, it acts by directly binding to the δ subunit. Several studies using ganaxolone show promising results, both in rodent seizure models [120–122] as well as in clinical studies [123]. In patients with infantile spasms, seizure frequency was decreased by at least 50 % in one third of the patients [124]. The frequency of occurrence and type of side effects were comparable to classic antiepileptic drugs. Currently, ganoxalone is investigated as an adjunctive treatment in patients with drug-resistant, partial onset seizures (trial identifier NCT01963208).

Also, the anesthetic 4,5,6,7-tetrahydroisoxazolo(5,4-c)pyridin-3-ol (THIP, gaboxadol) agonizes the δ subunit. THIP can reduce spikes in vitro and in vivo [125, 126]. In a clinical study with a small sample size, a trend toward seizure reduction was demonstrated [127]. However, there is conflicting evidence showing no effect on epileptiform activity [128, 129]. Other anesthetics, which selectively target the δ subunit are alphaxalone and propofol. Due to its anesthetic effects, these drugs are not suitable for the treatment of epilepsy.

A relatively new compound binding selectively to the δ subunit is the GABA agonist DS-1 and an enhancer DS-2. DS-2 has been shown to increase tonic currents in the thalamic neurons in vitro [130].

### Challenges of Enhancing Tonic Signaling

The development of AEDs aimed at tonic receptors is not straightforward due to several reasons. First of all, the long-term use of tonic GABA_A_R agonists, in particular neurosteroids, can lead to a downregulation of α4 and δ subunits. The elimination of the binding site by downregulation ceases the potential long-term benefits of this strategy. Furthermore, AEDs can have side effects. Side effects depend on the concentration of the compound and the distribution in the central nervous system. However, one advantage of extrasynaptic GABA_A_Rs is their cell type and brain region-specific subunit composition. The advantage of this heterogeneity of receptors is that all subunits differ in their affinity for agonists and antagonists. The aspecificity of drugs can therefore be partially overcome by using different dosages of AEDs. For instance, δ subunit containing receptors have the highest affinity for GABA.

Furthermore, compensatory alterations in other cells or brain regions could be a cause of ineffectiveness of AEDs. For instance, increasing GABA concentrations through inhibition of GAT-3 is not effective in the hippocampus due to the compensatory action of GAT-1. In the thalamus, however, GAT-3 inhibition increases tonic signaling. Related to these region-specific effects is the fact that certain types of epilepsy appear to be more suitable targets for tonic GABA modulation. For instance, in the absence of seizures, tonic inhibition in the thalamus is upregulated rather than downregulated [76]. Enhancing tonic signaling triggers slow wave discharges—a hallmark of this type of epilepsy—and consequently aggravates seizures [131]. In this light, it becomes plausible that an increase in tonic inhibition particularly in the thalamus causes absence-like side effects.

It is important to realize that an increase in extracellular GABA has consequences for synaptic receptors and receptors on which GABA acts as a (partial) ligand (i.e., GABA_B_Rs). In this regard, it might be sometimes difficult to disentangle whether the specific increase in tonic currents or a gross enhancement in the inhibitory tone causes antiepileptiform effects.

### Future Directions for the Development of Tonic AEDs

To overcome the challenges described above, it is necessary to reveal the complex network effects of tonic signaling and develop more specific and targeted drugs. By targeting specific subunits, one could make use of the particular pharmacokinetic properties such as affinity, channel opening time, refractory period, etc., which would allow fine-tuning of neuronal activity in the presence of varying concentrations of antagonist or agonists [23, 24, 88, 132].

Potential new specific strategies are evolving. An example of a cell-type specific therapy is optogenetics [133]. With optogenetics, certain genetically manipulated neurons are inhibited or excited by light. If one succeeds in selectively inhibiting the principal neuron only in the epileptogenic zone or increasing the activity of interneurons, this would potentially create new treatment opportunities. In the case of epilepsy, it could be beneficial to increase the release of GABA and therefore increase ambient GABA concentrations, which, in turn, can activate extrasynaptic receptors. Considering the important buffering capacity of astroglia cells, those might also form an attractive target with regard to regulating the concentration of extracellular GABA.

Additionally, by using cell-specific approaches, side effects could be limited. Also, designer receptors exclusively activated by designer drugs (DREADDs) might evolve as future treatment strategy [134]. With DREADDs, receptors are administered to the central nervous system via a viral vector into specific cell types. A specific oral drug activates these receptors. In fact, in epilepsy, there are two options with regard to DREADDs [135]. On one hand, inserting the receptor into the presynaptic gabaergic terminal and activating it by the oral drug could increase presynaptic GABA release and therefore augment not only tonic but also phasic inhibition. On the other hand, the precise activation of certain interneuron populations in the epileptogenic zone might contribute to a higher excitation threshold of the principal neuron, which, in turn, might be beneficial at the network level. The advantage of this procedure is the specificity of the treatment and fact that it is less invasive compared to optogenetic techniques.

Another strategy might be to interfere with receptor trafficking. A potential approach would be to enhance tonic signaling by increasing receptor expression and anchoring. This could be achieved by stimulating de novo synthesis or by promoting the migration of receptors from the synaptic to the extrasynaptic site. A potential consequence might be the loss of synaptic receptors, which, in turn, leads to the loss of phasic inhibition, which is detrimental to epilepsy. Next to increasing de novo synthesis, shuttling of ready-made receptors to the cell surface could be enhanced.

Lastly, it might be desirable to improve anchoring at the extrasynaptic side. In this regard, anchoring proteins such as radixin might play a crucial role. Whereas disrupting certain protein-protein interactions becomes more feasible by using virus-derived proteins [136, 137], enhancing these interactions is more difficult.

## Conclusion

Tonic inhibition is maintained in epilepsy and therefore serves as an attractive substrate for interventions. Drugs aimed at tonic GABA signaling, such as ganoxalone, show promising anticonvulsive results in rodent studies, but they trigger side effects as well. Future detailed knowledge about receptor trafficking and particular changes in specific brain regions will contribute to more rational drug design leading to more potent drugs with fewer side effects.

## Abbreviations

AED: Antiepileptic drug
CA: Cornu ammonis region
DG: Hippocampal dentate gyrus
GABARAP: γ-Aminobutyric type A receptor-associated protein
GABA_A_R: γ-Aminobutyric type A receptor
IPSP: Inhibitory postsynaptic potential
PSD: Postsynaptic density
TLE: Temporal lobe epilepsy

## References

[1] World Health Organization (2012) Epilepsy Fact Sheet http://www.who.int/mediacentre/factsheets/fs999/en/. Accessed 30th november 2014

[2] Wiebe S (2000). Epidemiology of temporal lobe epilepsy. Can J Neurol Sci Le J Can Sci Neurol.

[3] Kwan P, Brodie MJ (2000). Early identification of refractory epilepsy. N Engl J Med.

[4] Kwan P, Arzimanoglou A, Berg AT, Brodie MJ, Allen Hauser W, Mathern G, Moshe SL, Perucca E (2010). Definition of drug resistant epilepsy: consensus proposal by the ad hoc Task Force of the ILAE Commission on Therapeutic Strategies. Epilepsia.

[5] Farrant M, Nusser Z (2005). Variations on an inhibitory theme: phasic and tonic activation of GABA(A) receptors. Nat Rev Neurosci.

[6] Glykys J, Mody I (2007). Activation of GABAA receptors: views from outside the synaptic cleft. Neuron.

[7] Caraiscos VB, Elliott EM, You-Ten KE, Cheng VY, Belelli D, Newell JG, Jackson MF, Lambert JJ (2004). Tonic inhibition in mouse hippocampal CA1 pyramidal neurons is mediated by alpha5 subunit-containing gamma-aminobutyric acid type A receptors. Proc Natl Acad Sci U S A.

[8] Semyanov A, Walker MC, Kullmann DM, Silver RA (2004). Tonically active GABA A receptors: modulating gain and maintaining the tone. Trends Neurosci.

[9] Pavlov I, Savtchenko LP, Kullmann DM, Semyanov A, Walker MC (2009). Outwardly rectifying tonically active GABAA receptors in pyramidal cells modulate neuronal offset, not gain. J Neurosci Off J Soc Neurosci.

[10] Holter NI, Zylla MM, Zuber N, Bruehl C, Draguhn A (2010). Tonic GABAergic control of mouse dentate granule cells during postnatal development. Eur J Neurosci.

[11] Mann EO, Mody I (2010). Control of hippocampal gamma oscillation frequency by tonic inhibition and excitation of interneurons. Nat Neurosci.

[12] Martin LJ, Zurek AA, MacDonald JF, Roder JC, Jackson MF, Orser BA (2010). Alpha5GABAA receptor activity sets the threshold for long-term potentiation and constrains hippocampus-dependent memory. J Neurosci Off J Soc Neurosci.

[13] Duveau V, Laustela S, Barth L, Gianolini F, Vogt KE, Keist R, Chandra D, Homanics GE (2011). Spatiotemporal specificity of GABAA receptor-mediated regulation of adult hippocampal neurogenesis. Eur J Neurosci.

[14] Ge S, Goh EL, Sailor KA, Kitabatake Y, Ming GL, Song H (2006). GABA regulates synaptic integration of newly generated neurons in the adult brain. Nature.

[15] Mody I, Pearce RA (2004). Diversity of inhibitory neurotransmission through GABA(A) receptors. Trends Neurosci.

[16] Luscher B, Keller CA (2004). Regulation of GABAA receptor trafficking, channel activity, and functional plasticity of inhibitory synapses. Pharmacol Ther.

[17] Olsen RW, Sieghart W (2008). International Union of Pharmacology. LXX. Subtypes of gamma-aminobutyric acid(A) receptors: classification on the basis of subunit composition, pharmacology, and function. Update. Pharmacol Rev.

[18] Rudolph U, Mohler H (2004). Analysis of GABAA receptor function and dissection of the pharmacology of benzodiazepines and general anesthetics through mouse genetics. Annu Rev Pharmacol Toxicol.

[19] Lee V, Maguire J (2014). The impact of tonic GABAA receptor-mediated inhibition on neuronal excitability varies across brain region and cell type. Front Neural Circ.

[20] Sieghart W, Sperk G (2002). Subunit composition, distribution and function of GABA(A) receptor subtypes. Curr Top Med Chem.

[21] Wei W, Zhang N, Peng Z, Houser CR, Mody I (2003). Perisynaptic localization of delta subunit-containing GABA(A) receptors and their activation by GABA spillover in the mouse dentate gyrus. J Neurosci: Off J Soc Neurosci.

[22] Nusser Z, Sieghart W, Somogyi P (1998). Segregation of different GABAA receptors to synaptic and extrasynaptic membranes of cerebellar granule cells. J Neurosci: Off J Soc Neurosci.

[23] Glykys J, Mody I (2006). Hippocampal network hyperactivity after selective reduction of tonic inhibition in GABA A receptor alpha5 subunit-deficient mice. J Neurophysiol.

[24] Semyanov A, Walker MC, Kullmann DM (2003). GABA uptake regulates cortical excitability via cell type-specific tonic inhibition. Nat Neurosci.

[25] Stell BM, Brickley SG, Tang CY, Farrant M, Mody I (2003). Neuroactive steroids reduce neuronal excitability by selectively enhancing tonic inhibition mediated by delta subunit-containing GABAA receptors. Proc Natl Acad Sci U S A.

[26] Glykys J, Mann EO, Mody I (2008). Which GABA(A) receptor subunits are necessary for tonic inhibition in the hippocampus?. J Neurosci: Off J Soc Neurosci.

[27] Serwanski DR, Miralles CP, Christie SB, Mehta AK, Li X, De Blas AL (2006). Synaptic and nonsynaptic localization of GABAA receptors containing the alpha5 subunit in the rat brain. J Comp Neurol.

[28] Michels G, Moss SJ (2007). GABAA receptors: properties and trafficking. Crit Rev Biochem Mol Biol.

[29] Knoflach F, Benke D, Wang Y, Scheurer L, Luddens H, Hamilton BJ, Carter DB, Mohler H (1996). Pharmacological modulation of the diazepam-insensitive recombinant gamma-aminobutyric acidA receptors alpha 4 beta 2 gamma 2 and alpha 6 beta 2 gamma 2. Mol Pharmacol.

[30] Fisher JL, Macdonald RL (1997). Single channel properties of recombinant GABAA receptors containing gamma 2 or delta subtypes expressed with alpha 1 and beta 3 subtypes in mouse L929 cells. J Physiol.

[31] Bohme I, Rabe H, Luddens H (2004). Four amino acids in the alpha subunits determine the gamma-aminobutyric acid sensitivities of GABAA receptor subtypes. J Biol Chem.

[32] Minier F, Sigel E (2004). Positioning of the alpha-subunit isoforms confers a functional signature to gamma-aminobutyric acid type A receptors. Proc Natl Acad Sci U S A.

[33] Brown N, Kerby J, Bonnert TP, Whiting PJ, Wafford KA (2002). Pharmacological characterization of a novel cell line expressing human alpha(4)beta(3)delta GABA(A) receptors. Br J Pharmacol.

[34] Rossi DJ, Hamann M (1998). Spillover-mediated transmission at inhibitory synapses promoted by high affinity alpha6 subunit GABA(A) receptors and glomerular geometry. Neuron.

[35] Otis TS, Staley KJ, Mody I (1991). Perpetual inhibitory activity in mammalian brain slices generated by spontaneous GABA release. Brain Res.

[36] Barbour B, Hausser M (1997). Intersynaptic diffusion of neurotransmitter. Trends Neurosci.

[37] Wall MJ, Usowicz MM (1997). Development of action potential-dependent and independent spontaneous GABAA receptor-mediated currents in granule cells of postnatal rat cerebellum. Eur J Neurosci.

[38] Rossi DJ, Hamann M, Attwell D (2003). Multiple modes of GABAergic inhibition of rat cerebellar granule cells. J Physiol.

[39] Yoon BE, Lee CJ (2014). GABA as a rising gliotransmitter. Frontiers Neural Circ.

[40] Zilberter Y, Kaiser KM, Sakmann B (1999). Dendritic GABA release depresses excitatory transmission between layer 2/3 pyramidal and bitufted neurons in rat neocortex. Neuron.

[41] Le Meur K, Mendizabal-Zubiaga J, Grandes P, Audinat E (2012). GABA release by hippocampal astrocytes. Front Comput Neurosci.

[42] Kozlov AS, Angulo MC, Audinat E, Charpak S (2006). Target cell-specific modulation of neuronal activity by astrocytes. Proc Natl Acad Sci U S A.

[43] Jimenez-Gonzalez C, Pirttimaki T, Cope DW, Parri HR (2011). Non-neuronal, slow GABA signalling in the ventrobasal thalamus targets delta-subunit-containing GABA(A) receptors. Eur J Neurosci.

[44] Lee S, Yoon BE, Berglund K, Oh SJ, Park H, Shin HS, Augustine GJ, Lee CJ (2010). Channel-mediated tonic GABA release from glia. Science.

[45] Attwell D, Barbour B, Szatkowski M (1993). Nonvesicular release of neurotransmitter. Neuron.

[46] Cammack JN, Rakhilin SV, Schwartz EA (1994). A GABA transporter operates asymmetrically and with variable stoichiometry. Neuron.

[47] Levi G, Raiteri M (1993). Carrier-mediated release of neurotransmitters. Trends Neurosci.

[48] Lu CC, Hilgemann DW (1999). GAT1 (GABA:Na+:Cl-) cotransport function. Kinetic studies in giant Xenopus oocyte membrane patches. J Gen Physiol.

[49] O’Malley DM, Sandell JH, Masland RH (1992). Co-release of acetylcholine and GABA by the starburst amacrine cells. J Neurosci: Off J Soc Neurosci.

[50] Pin JP, Bockaert J (1989). Two distinct mechanisms, differentially affected by excitatory amino acids, trigger GABA release from fetal mouse striatal neurons in primary culture. J Neurosci: Off J Soc Neurosci.

[51] Schwartz EA (1987). Depolarization without calcium can release gamma-aminobutyric acid from a retinal neuron. Science.

[52] Wlodarczyk AI, Sylantyev S, Herd MB, Kersante F, Lambert JJ, Rusakov DA, Linthorst AC, Semyanov A (2013). GABA-independent GABAA receptor openings maintain tonic currents. J Neurosci: Off J Soc Neurosci.

[53] McCartney MR, Deeb TZ, Henderson TN, Hales TG (2007). Tonically active GABAA receptors in hippocampal pyramidal neurons exhibit constitutive GABA-independent gating. Mol Pharmacol.

[54] Sigel E, Baur R, Malherbe P, Mohler H (1989). The rat beta 1-subunit of the GABAA receptor forms a picrotoxin-sensitive anion channel open in the absence of GABA. FEBS Lett.

[55] Maksay G, Thompson SA, Wafford KA (2003). The pharmacology of spontaneously open alpha 1 beta 3 epsilon GABA A receptor-ionophores. Neuropharmacology.

[56] Lindquist CE, Dalziel JE, Cromer BA, Birnir B (2004). Penicillin blocks human alpha 1 beta 1 and alpha 1 beta 1 gamma 2S GABAA channels that open spontaneously. Eur J Pharmacol.

[57] Birnir B, Everitt AB, Lim MS, Gage PW (2000). Spontaneously opening GABA(A) channels in CA1 pyramidal neurones of rat hippocampus. J Membrane Biol.

[58] Maguire J, Mody I (2008). GABA(A)R plasticity during pregnancy: relevance to postpartum depression. Neuron.

[59] Maguire J, Mody I (2009). Steroid hormone fluctuations and GABA(A)R plasticity. Psychoneuroendocrinology.

[60] Tao W, Higgs MH, Spain WJ, Ransom CB (2013). Postsynaptic GABAB receptors enhance extrasynaptic GABAA receptor function in dentate gyrus granule cells. J Neurosci Off J Soc Neurosci.

[61] Connelly WM, Fyson SJ, Errington AC, McCafferty CP, Cope DW, Di Giovanni G, Crunelli V (2013). GABAB receptors regulate extrasynaptic GABAA receptors. J Neurosci Off J Soc Neurosci.

[62] Scanziani M, Gahwiler BH, Thompson SM (1991). Paroxysmal inhibitory potentials mediated by GABAB receptors in partially disinhibited rat hippocampal slice cultures. J Physiol.

[63] During MJ, Spencer DD (1993). Extracellular hippocampal glutamate and spontaneous seizure in the conscious human brain. Lancet.

[64] Barbaresi P (2007). Cellular and subcellular localization of the GABA(B) receptor 1a/b subunit in the rat periaqueductal gray matter. J Comp Neurol.

[65] Andersson M, Blomstrand F, Hanse E (2007). Astrocytes play a critical role in transient heterosynaptic depression in the rat hippocampal CA1 region. J Physiol.

[66] Tretter V, Mukherjee J, Maric HM, Schindelin H, Sieghart W, Moss SJ (2012). Gephyrin, the enigmatic organizer at GABAergic synapses. Front Cell Neurosci.

[67] Jacob TC, Moss SJ, Jurd R (2008). GABA(A) receptor trafficking and its role in the dynamic modulation of neuronal inhibition. Nat Rev Neurosci.

[68] Loebrich S, Bahring R, Katsuno T, Tsukita S, Kneussel M (2006). Activated radixin is essential for GABAA receptor alpha5 subunit anchoring at the actin cytoskeleton. EMBO J.

[69] Thomas P, Mortensen M, Hosie AM, Smart TG (2005). Dynamic mobility of functional GABAA receptors at inhibitory synapses. Nat Neurosci.

[70] Bogdanov Y, Michels G, Armstrong-Gold C, Haydon PG, Lindstrom J, Pangalos M, Moss SJ (2006). Synaptic GABAA receptors are directly recruited from their extrasynaptic counterparts. EMBO J.

[71] Petrini EM, Marchionni I, Zacchi P, Sieghart W, Cherubini E (2004). Clustering of extrasynaptic GABA(A) receptors modulates tonic inhibition in cultured hippocampal neurons. J Biol Chem.

[72] Chen L, Wang H, Vicini S, Olsen RW (2000). The gamma-aminobutyric acid type A (GABAA) receptor-associated protein (GABARAP) promotes GABAA receptor clustering and modulates the channel kinetics. Proc Natl Acad Sci U S A.

[73] Semyanov A (2003). Cell type specificity of GABA(A) receptor mediated signaling in the hippocampus. Curr Drug Targets CNS Neurol Disord.

[74] Brickley SG, Cull-Candy SG, Farrant M (1996). Development of a tonic form of synaptic inhibition in rat cerebellar granule cells resulting from persistent activation of GABAA receptors. J Physiol.

[75] Song I, Savtchenko L, Semyanov A (2011). Tonic excitation or inhibition is set by GABA(A) conductance in hippocampal interneurons. Nat Commun.

[76] Cope DW, Di Giovanni G, Fyson SJ, Orban G, Errington AC, Lorincz ML, Gould TM, Carter DA (2009). Enhanced tonic GABAA inhibition in typical absence epilepsy. Nat Med.

[77] Clarkson AN, Huang BS, Macisaac SE, Mody I, Carmichael ST (2010). Reducing excessive GABA-mediated tonic inhibition promotes functional recovery after stroke. Nature.

[78] Dibbens LM, Feng HJ, Richards MC, Harkin LA, Hodgson BL, Scott D, Jenkins M, Petrou S (2004). GABRD encoding a protein for extra- or peri-synaptic GABAA receptors is a susceptibility locus for generalized epilepsies. Hum Mol Genet.

[79] Feng HJ, Kang JQ, Song L, Dibbens L, Mulley J, Macdonald RL (2006). Delta subunit susceptibility variants E177A and R220H associated with complex epilepsy alter channel gating and surface expression of alpha4beta2delta GABAA receptors. J Neurosci: Off J Soc Neurosci.

[80] Eugene E, Depienne C, Baulac S, Baulac M, Fritschy JM, Le Guern E, Miles R, Poncer JC (2007). GABA(A) receptor gamma 2 subunit mutations linked to human epileptic syndromes differentially affect phasic and tonic inhibition. J Neurosci: Off J Soc Neurosci.

[81] Feng Y, Kapornai K, Kiss E, Tamas Z, Mayer L, Baji I, Daroczi G, Benak I (2010). Association of the GABRD gene and childhood-onset mood disorders. Genes Brain Behav.

[82] Schwarzer C, Sperk G (1995). Hippocampal granule cells express glutamic acid decarboxylase-67 after limbic seizures in the rat. Neuroscience.

[83] Fritschy JM, Kiener T, Bouilleret V, Loup F (1999). GABAergic neurons and GABA(A)-receptors in temporal lobe epilepsy. Neurochem Int.

[84] Bouilleret V, Loup F, Kiener T, Marescaux C, Fritschy JM (2000). Early loss of interneurons and delayed subunit-specific changes in GABA(A)-receptor expression in a mouse model of mesial temporal lobe epilepsy. Hippocampus.

[85] Drexel M, Kirchmair E, Sperk G (2013). Changes in the expression of GABAA receptor subunit mRNAs in parahippocampal areas after kainic acid induced seizures. Front Neural Circ.

[86] Nishimura T, Schwarzer C, Gasser E, Kato N, Vezzani A, Sperk G (2005). Altered expression of GABA(A) and GABA(B) receptor subunit mRNAs in the hippocampus after kindling and electrically induced status epilepticus. Neuroscience.

[87] Tsunashima K, Schwarzer C, Kirchmair E, Sieghart W, Sperk G (1997). GABA(A) receptor subunits in the rat hippocampus III: altered messenger RNA expression in kainic acid-induced epilepsy. Neuroscience.

[88] Scimemi A, Semyanov A, Sperk G, Kullmann DM, Walker MC (2005). Multiple and plastic receptors mediate tonic GABAA receptor currents in the hippocampus. J Neurosci: Off J Soc Neurosci.

[89] Zhang N, Wei W, Mody I, Houser CR (2007). Altered localization of GABA(A) receptor subunits on dentate granule cell dendrites influences tonic and phasic inhibition in a mouse model of epilepsy. J Neurosci: Off J Soc Neurosci.

[90] Peng Z, Huang CS, Stell BM, Mody I, Houser CR (2004). Altered expression of the delta subunit of the GABAA receptor in a mouse model of temporal lobe epilepsy. J Neurosci: Off J Soc Neurosci.

[91] Rajasekaran K, Joshi S, Sun C, Mtchedlishvilli Z, Kapur J (2010). Receptors with low affinity for neurosteroids and GABA contribute to tonic inhibition of granule cells in epileptic animals. Neurobiol Dis.

[92] Scimemi A, Andersson A, Heeroma JH, Strandberg J, Rydenhag B, McEvoy AW, Thom M, Asztely F (2006). Tonic GABA(A) receptor-mediated currents in human brain. Eur J Neurosci.

[93] Goodkin HP, Joshi S, Mtchedlishvili Z, Brar J, Kapur J (2008). Subunit-specific trafficking of GABA(A) receptors during status epilepticus. J Neurosci Off J Soc Neurosci.

[94] Naylor DE, Liu H, Wasterlain CG (2005). Trafficking of GABA(A) receptors, loss of inhibition, and a mechanism for pharmacoresistance in status epilepticus. J Neurosci Off J Soc Neurosci.

[95] Sperk G, Schwarzer C, Heilman J, Furtinger S, Reimer RJ, Edwards RH, Nelson N (2003). Expression of plasma membrane GABA transporters but not of the vesicular GABA transporter in dentate granule cells after kainic acid seizures. Hippocampus.

[96] Mathern GW, Mendoza D, Lozada A, Pretorius JK, Dehnes Y, Danbolt NC, Nelson N, Leite JP (1999). Hippocampal GABA and glutamate transporter immunoreactivity in patients with temporal lobe epilepsy. Neurology.

[97] Andre V, Marescaux C, Nehlig A, Fritschy JM (2001). Alterations of hippocampal GAbaergic system contribute to development of spontaneous recurrent seizures in the rat lithium-pilocarpine model of temporal lobe epilepsy. Hippocampus.

[98] Esclapez M, Houser CR (1999). Up-regulation of GAD65 and GAD67 in remaining hippocampal GABA neurons in a model of temporal lobe epilepsy. J Comp Neurol.

[99] Freichel C, Potschka H, Ebert U, Brandt C, Loscher W (2006). Acute changes in the neuronal expression of GABA and glutamate decarboxylase isoforms in the rat piriform cortex following status epilepticus. Neuroscience.

[100] Ding R, Asada H, Obata K (1998). Changes in extracellular glutamate and GABA levels in the hippocampal CA3 and CA1 areas and the induction of glutamic acid decarboxylase-67 in dentate granule cells of rats treated with kainic acid. Brain Res.

[101] Kash SF, Johnson RS, Tecott LH, Noebels JL, Mayfield RD, Hanahan D, Baekkeskov S (1997). Epilepsy in mice deficient in the 65-kDa isoform of glutamic acid decarboxylase. Proc Natl Acad Sci U S A.

[102] Pavlov I, Walker MC (2013). Tonic GABA(A) receptor-mediated signalling in temporal lobe epilepsy. Neuropharmacology.

[103] Schwarzer C, Tsunashima K, Wanzenbock C, Fuchs K, Sieghart W, Sperk G (1997). GABA(A) receptor subunits in the rat hippocampus II: altered distribution in kainic acid-induced temporal lobe epilepsy. Neuroscience.

[104] Zhan RZ, Nadler JV (2009). Enhanced tonic GABA current in normotopic and hilar ectopic dentate granule cells after pilocarpine-induced status epilepticus. J Neurophysiol.

[105] Lee B, Lee H, Nam YR, Oh JH, Cho YH, Chang JW (2005). Enhanced expression of glutamate decarboxylase 65 improves symptoms of rat parkinsonian models. Gene Ther.

[106] Emborg ME, Carbon M, Holden JE, During MJ, Ma Y, Tang C, Moirano J, Fitzsimons H (2007). Subthalamic glutamic acid decarboxylase gene therapy: changes in motor function and cortical metabolism. J Cereb Blood Flow Metab: Off J Int Soc Cereb Blood Flow Metab.

[107] Kaplitt MG, Feigin A, Tang C, Fitzsimons HL, Mattis P, Lawlor PA, Bland RJ, Young D (2007). Safety and tolerability of gene therapy with an adeno-associated virus (AAV) borne GAD gene for Parkinson’s disease: an open label, phase I trial. Lancet.

[108] LeWitt PA, Guttman M, Tetrud JW, Tuite PJ, Mori A, Chaikin P, Sussman NM, Group USS (2008). Adenosine A2A receptor antagonist istradefylline (KW-6002) reduces “off” time in Parkinson’s disease: a double-blind, randomized, multicenter clinical trial (6002-US-005). Ann Neurol.

[109] Grove J, Fozard JR, Mamont PS (1981). Assay of alpha-difluoromethylornithine in body fluids and tissues by automatic amino-acid analysis. J Chromatogr.

[110] Mumford JP, Dam M (1989). Meta-analysis of European placebo controlled studies of vigabatrin in drug resistant epilepsy. Br J Clin Pharmacol.

[111] Jung MJ, Lippert B, Metcalf BW, Bohlen P, Schechter PJ (1977). gamma-Vinyl GABA (4-amino-hex-5-enoic acid), a new selective irreversible inhibitor of GABA-T: effects on brain GABA metabolism in mice. J Neurochem.

[112] Angehagen M, Ben-Menachem E, Ronnback L, Hansson E (2003). Novel mechanisms of action of three antiepileptic drugs, vigabatrin, tiagabine, and topiramate. Neurochem Res.

[113] Leach JP, Sills GJ, Majid A, Butler E, Carswell A, Thompson GG, Brodie MJ (1996). Effects of tiagabine and vigabatrin on GABA uptake into primary cultures of rat cortical astrocytes. Seizure.

[114] Duffy S, Nguyen PV, Baker GB (2004). Phenylethylidenehydrazine, a novel GABA-transaminase inhibitor, reduces epileptiform activity in rat hippocampal slices. Neuroscience.

[115] Loscher W (1980). A comparative study of the pharmacology of inhibitors of GABA-metabolism. Naunyn Schmiedeberg’s Arch Pharmacol.

[116] Borden LA, Murali Dhar TG, Smith KE, Weinshank RL, Branchek TA, Gluchowski C (1994). Tiagabine, SK&F 89976-A, CI-966, and NNC-711 are selective for the cloned GABA transporter GAT-1. Eur J Pharmacol.

[117] Fink-Jensen A, Suzdak PD, Swedberg MD, Judge ME, Hansen L, Nielsen PG (1992). The gamma-aminobutyric acid (GABA) uptake inhibitor, tiagabine, increases extracellular brain levels of GABA in awake rats. Eur J Pharmacol.

[118] Wilby J, Kainth A, Hawkins N, Epstein D, McIntosh H, McDaid C, Mason A, Golder S (2005). Clinical effectiveness, tolerability and cost-effectiveness of newer drugs for epilepsy in adults: a systematic review and economic evaluation. Health Technol Assess.

[119] Kellinghaus C, Loddenkemper T, Weitemeyer L, Ludemann P (2001). Experience with tiagabine in the clinical practice; new insights as to the efficacy and safety profile. Nervenarzt.

[120] Bialer M, White HS (2010). Key factors in the discovery and development of new antiepileptic drugs. Nat Rev Drug Discov.

[121] Reddy DS, Rogawski MA (2012) Neurosteroids—endogenous regulators of seizure susceptibility and role in the treatment of epilepsy. In: Noebels JL, Avoli M, Rogawski MA, Olsen RW, Delgado-Escueta AV (eds) Jasper’s Basic Mechanisms of the Epilepsies. 4th edn., Bethesda (MD) 22787590

[122] Reddy DS, Rogawski MA (2010). Ganaxolone suppression of behavioral and electrographic seizures in the mouse amygdala kindling model. Epilepsy Res.

[123] Bialer M, Johannessen SI, Levy RH, Perucca E, Tomson T, White HS (2013). Progress report on new antiepileptic drugs: a summary of the Eleventh Eilat Conference (EILAT XI). Epilepsy Res.

[124] Kerrigan JF, Shields WD, Nelson TY, Bluestone DL, Dodson WE, Bourgeois BF, Pellock JM, Morton LD (2000). Ganaxolone for treating intractable infantile spasms: a multicenter, open-label, add-on trial. Epilepsy Res.

[125] Wan L, Liu X, Wu Z, Ren W, Kong S, Dargham RA, Cheng L, Wang Y (2014). Activation of extrasynaptic GABAA receptors inhibits cyclothiazide-induced epileptiform activity in hippocampal CA1 neurons. Neurosci Bull.

[126] Sun Y, Wu Z, Kong S, Jiang D, Pitre A, Wang Y, Chen G (2013). Regulation of epileptiform activity by two distinct subtypes of extrasynaptic GABAA receptors. Mole Brain.

[127] Petersen HR, Jensen I, Dam M (1983). THIP: a single-blind controlled trial in patients with epilepsy. Acta Neurol Scand.

[128] Hansen SL, Sperling BB, Sanchez C (2004). Anticonvulsant and antiepileptogenic effects of GABAA receptor ligands in pentylenetetrazole-kindled mice. Prog Neuro-Psychopharmacol Biol Psychiatry.

[129] Loscher W, Schwark WS (1985). Evaluation of different GABA receptor agonists in the kindled amygdala seizure model in rats. Exp Neurol.

[130] Wafford KA, van Niel MB, Ma QP, Horridge E, Herd MB, Peden DR, Belelli D, Lambert JJ (2009). Novel compounds selectively enhance delta subunit containing GABA A receptors and increase tonic currents in thalamus. Neuropharmacology.

[131] Crunelli V, Leresche N, Cope DW (2012) GABA-A receptor function in typical absence seizures. In: Noebels JL, Avoli M, Rogawski MA, Olsen RW, Delgado-Escueta AV (eds) Jasper’s Basic Mechanisms of the Epilepsies. 4th edn., Bethesda (MD) 22787597

[132] Prenosil GA, Schneider Gasser EM, Rudolph U, Keist R, Fritschy JM, Vogt KE (2006). Specific subtypes of GABAA receptors mediate phasic and tonic forms of inhibition in hippocampal pyramidal neurons. J Neurophysiol.

[133] Krook-Magnuson E, Soltesz I (2015). Beyond the hammer and the scalpel: selective circuit control for the epilepsies. Nat Neurosci.

[134] Aston-Jones G, Deisseroth K (2013). Recent advances in optogenetics and pharmacogenetics. Brain Res.

[135] Katzel D, Nicholson E, Schorge S, Walker MC, Kullmann DM (2014). Chemical-genetic attenuation of focal neocortical seizures. Nat Commun.

[136] Aarts M, Liu Y, Liu L, Besshoh S, Arundine M, Gurd JW, Wang YT, Salter MW (2002). Treatment of ischemic brain damage by perturbing NMDA receptor-PSD-95 protein interactions. Science.

[137] Cook DJ, Teves L, Tymianski M (2012). Treatment of stroke with a PSD-95 inhibitor in the gyrencephalic primate brain. Nature.

